# Synaptotoxicity of Alzheimer Beta Amyloid Can Be Explained by Its Membrane Perforating Property

**DOI:** 10.1371/journal.pone.0011820

**Published:** 2010-07-27

**Authors:** Fernando J. Sepulveda, Jorge Parodi, Robert W. Peoples, Carlos Opazo, Luis G. Aguayo

**Affiliations:** 1 Laboratory of Neurophysiology, Department of Physiology, University of Concepción, Concepción, Chile; 2 Laboratory of Neurobiometals, Department of Physiology, University of Concepción, Concepción, Chile; 3 Department of Biomedical Sciences, Marquette University, Milwaukee, Wisconsin, United States of America; 4 Centro de Investigación Avanzada en Educación, University of Concepción, Concepción, Chile; University of Nebraska, United States of America

## Abstract

The mechanisms that induce Alzheimer's disease (AD) are largely unknown thereby deterring the development of disease-modifying therapies. One working hypothesis of AD is that Aβ excess disrupts membranes causing pore formation leading to alterations in ionic homeostasis. However, it is largely unknown if this also occurs in native brain neuronal membranes. Here we show that similar to other pore forming toxins, Aβ induces perforation of neuronal membranes causing an increase in membrane conductance, intracellular calcium and ethidium bromide influx. These data reveal that the target of Aβ is not another membrane protein, but that Aβ itself is the cellular target thereby explaining the failure of current therapies to interfere with the course of AD. We propose that this novel effect of Aβ could be useful for the discovery of anti AD drugs capable of blocking these “Aβ perforates”. In addition, we demonstrate that peptides that block Aβ neurotoxicity also slow or prevent the membrane-perforating action of Aβ.

## Introduction

Alzheimer's disease (AD) is a progressive and irreversible neurodegenerative brain disorder that leads to major debilitating cognitive deficits in the elderly. It is now believed that the cellular and molecular alterations that cause brain dysfunctions are slow in onset and that it probably takes several years to develop the full blown disease [1]. Surprisingly, the cellular and molecular mechanisms that induce AD are largely unknown, deterring the development of effective modifying or symptomatic therapies. Thus, attempts to alleviate and stop AD symptoms are actually based on compensating synaptic deficits and blocking intracellular signaling cascades [2]. However, the results are minor because at clinical stages the AD brain is already too deteriorated.

It is accepted that the toxic effects of Aβ, one etiological agent in AD, depend on dimer formation and subsequent oligomerization which include diverse structural forms [3]. Thus, blocking dimerization reduces aggregation and the ensuing peptide toxicity [4]. The working hypothesis of AD is that excess of Aβ either i) binds to membrane receptors affecting their functions [5], ii) interferes with signaling cascades [6]–[8] or iii) directly disrupts neuronal membranes causing pore formation leading to alterations in ionic homeostasis [9]. Although the latter is an attractive hypothesis because it could explain several effects of Aβ in brain neurons, it is largely unknown if this can also occur in native brain neuronal membranes. Additionally, the existence of this membrane phenomenon will reveal that the target of Aβ is not another membrane protein, but that Aβ itself is the cellular target and explain the failure to interfere with the course of AD. In agreement with this idea, atomic force microscopy (AFM) in lipid environments and molecular dynamic analysis have shown the presence of molecular entities with inner diameters in the 1.5–2.6 nm range [10], [11] which were similar to those generated by other peptidergic molecules known to form pores in cell membranes, such as amylin and α-synuclein [12].

For many years it has been recognized that several peptides with differing structures such as gramicidin, amphotericin and α-latrotoxin can alter membrane permeability after inducing pore formation [13], [14]. Additionally, it is known that antifungal antibiotics are toxic because they can attach to the cell wall and steadily disrupt permeability. Electrophysiologists have utilized gramicidin and amphotericin for more than 20 years to perforate cell membranes and record whole cell ionic currents with the patch clamp technique [13], [15], [16]. In the patch perforated mode, the membrane is disrupted thereby making holes that allow the continuous flow of ionic currents under the patch pipette. Here, we report that Aβ *has a rapid and potent perforating property in neuronal membranes*. We postulate that these perforations increase intracellular calcium leading to synaptic transmission failure [17]. Based on this membrane property, similar to gramicidin and amphotericin, we have defined Aβ as a perforating toxic agent, rather than a classical pore-forming agent.

## Results

### Aβ perforates hippocampal neuron membranes

**Figure 1:**
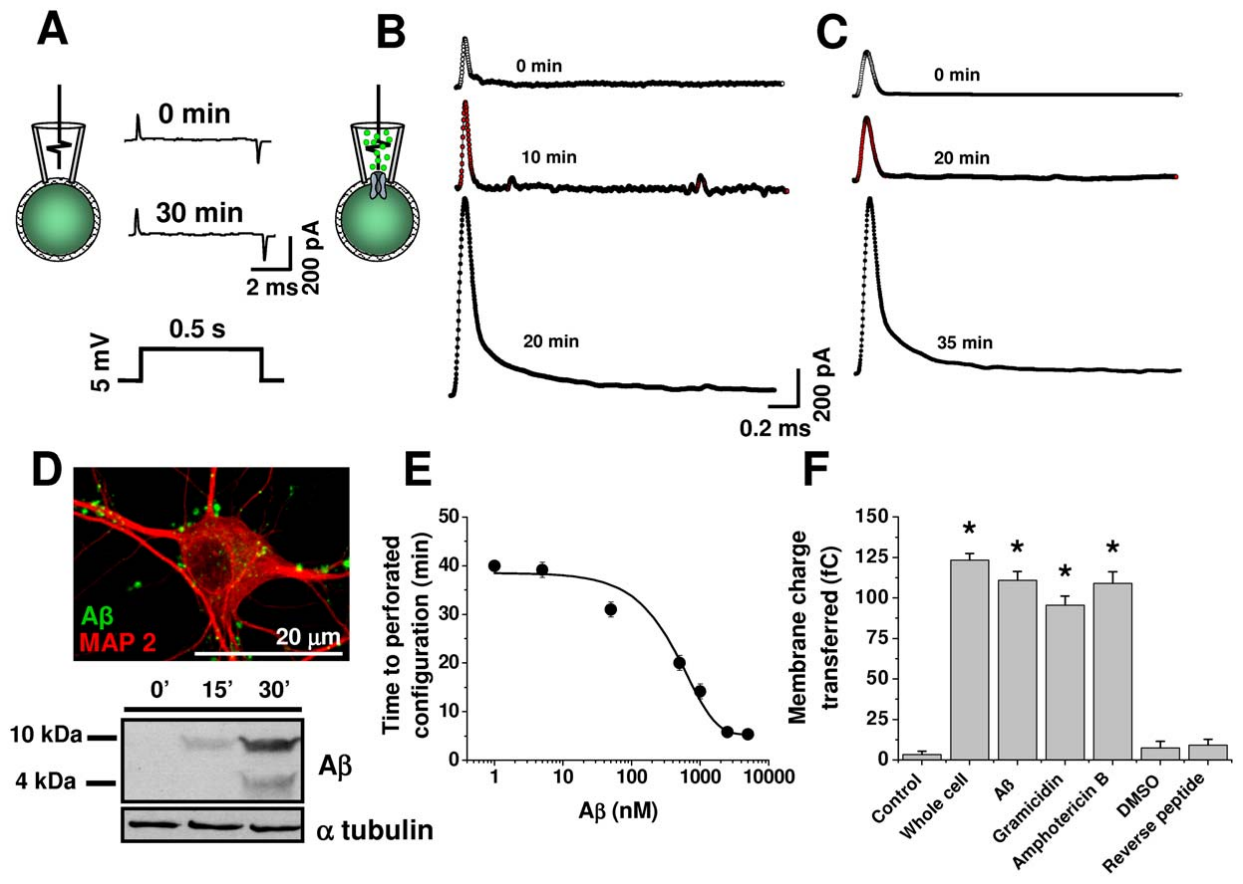
Aβ peptide induced formation of membrane perforation in hippocampal neurons. **A**, currents induced by a 5 mV depolarizing pulse recorded with control solution at two times in cell-attached mode. **B**, effect of application of 500 nM (2.2 µg/ml) Aβ via the patch pipette on capacitative membrane current. **C**, effect of gramicidin (100 µg/ml) on membrane current. **D**, confocal image shows a neuron stained for 15 minutes with 500 nM fluorescent Aβ. Western blot shows the time dependent association of low molecular weight oligomers with hippocampal cell membranes. **E**, effect of increasing Aβ concentrations on the time to establish the perforated configuration. **F**, effects of Aβ gramicidin, and amphotericin on the transferred membrane charge induced by 5 mV depolarization. Each point (mean ± SEM) was measured in a least 6 different hippocampal neurons. * denotes a P<0.05 (n = 6–7 neurons).

The cell attached mode of the patch clamp technique allows for stable measurements at the single molecule level. Thus, activation of most single voltage or ligand activated channel proteins can be adequately time resolved with open kinetics and complex cellular regulations [16], [18]. Alternatively, small peptides can produce minute disturbances in membrane stability causing a low resistance pathway under the membrane patch, known as a “perforated recording configuration” [19]. In this study, cell-attached recordings in hippocampal neurons were stable using a control solution in the patch pipette (i.e. >30 min). For example, the application of a 5 mV voltage pulse induced a stable, fast current arising from a partly compensated electrode capacitance (Fig. 1A). This current was markedly altered when 500 nM of pre-aggregated Aβ (see methods) was added into the patch pipette solution and allowed to diffuse to the underlying membrane (Fig. 1B). For example, the traces in figure 1B clearly show that the amplitude and time course of the capacitive current increased with time and this was similar to that induced by gramicidin (Fig. 1C). Fig. 1D obtained with a fluorescent form of Aβ and with Western blot analysis shows that the peptide was able to associate with neuronal membranes at times when it was producing membrane perforation (i.e. 15–30 min). The data also show that the time it took to form the perforated configuration by Aβ was dependent on the peptide concentration (Fig. 1E). For example, it took nearly 40 min to establish perforated recordings with 1 nM Aβ and less than 10 min with micromolar concentrations. Aβ (2.2 µg/ml, 500 nM) was more potent and rapid than gramicidin in forming the perforated configuration. However, the perforated configuration formation with gramicidin (100 µg/ml) can take more than 30 min [20]. Interestingly, Aβ_1–42_ produced similar effects in membrane charge and input resistance as those of Aβ_1–40_ (Fig. S1B,C). Furthermore, the Aβ-dependent actions were demonstrated by their blockade with the Aβ WO2 antibody that recognizes residues 1 to 5 of Aβ (Fig. S1B,C).

The analysis of the charge transferred during the capacitative response showed that the effect of Aβ was similar to those of gramicidin and amphotericin, two peptides commonly used to perforate neuron membranes (Fig. 1F). On the other hand, the effect of Aβ_1–40_ was not produced by the reverse Aβ peptide, supporting the idea that Aβ aggregation leads to membrane damage. Finally, the membrane currents induced by Aβ were very similar to those induced by positive pressure in the whole cell configuration, suggesting that they can transfer significant charge, equivalent to that induced by positive pressure, which is believed to completely burst the membrane under the patch pipette [18].

### Aβ displayed a “gramicidin-like” behavior in neuronal membranes

**Figure 2:**
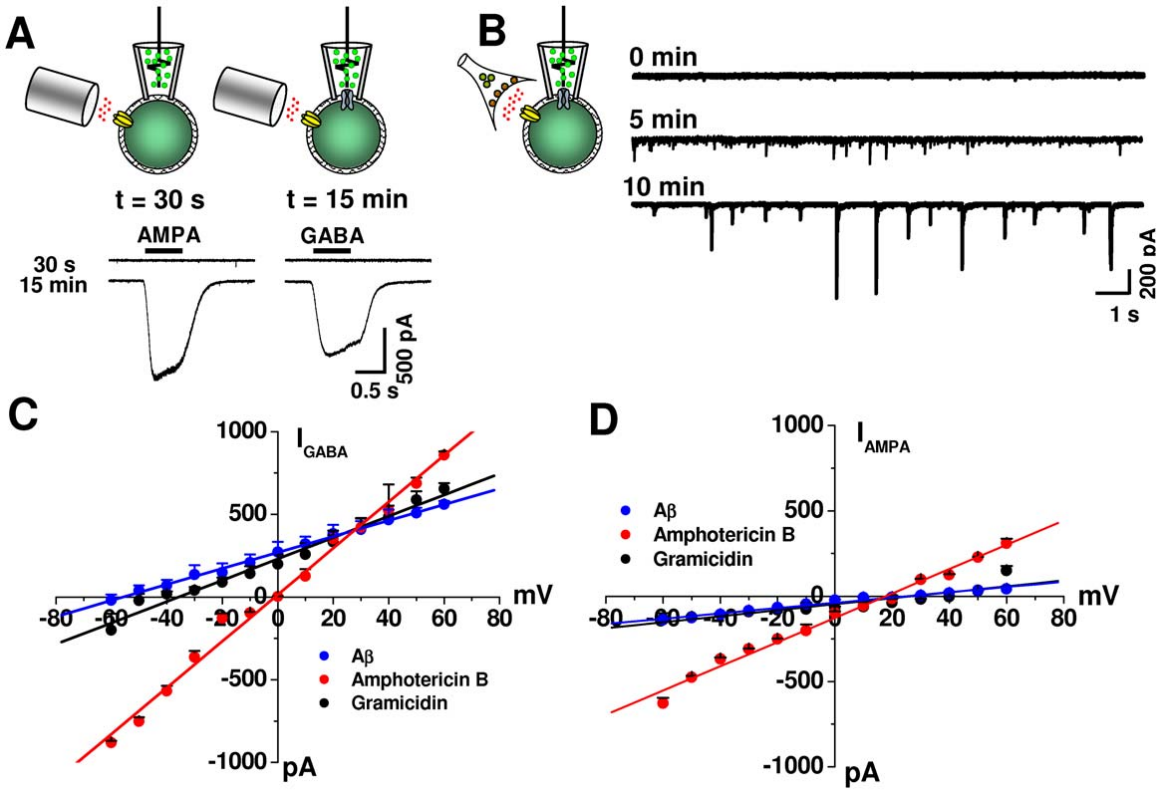
Aβ peptide produced cationic perforates comparable to gramicidin. **A**, currents were recorded using a cell-attached configuration at the beginning (30 s) and after 15 min of Aβ application via the patch pipette. AMPA and GABA (50 µM) applied to the extracellular membrane induced membrane currents after formation of membrane perforation. **B**, gradual appearance of synaptically mediated membrane currents after establishing the cell-attached conformation. **C**, GABA-induced anionic current-voltage relationships obtained during perforated mode with either gramicidin, amphotericin-B or Aβ. **D**, AMPA induced cationic current using gramicidin, amphotericin -B or Aβ. Each point (mean ± SEM) was measured in a least 5 different hippocampal neurons.

Peptides that perforate cell membranes can form pathways which are more or less selective to cations or anions [15], [16]. Gramicidin and amphotericin, for example, are used to record GABA_A_ and glutamatergic whole-cell currents, respectively, because while the former generates mainly cationic pores in the membrane, the latter is somewhat more selective for anions. Consistent with a time dependent membrane perforation process, the application of extracellular GABA or glutamate only 30 s after GΩ seal formation was unable to induce detectable membrane currents. This demonstrates the existence of a high resistance pathway between the membrane and the patch pipette containing 500 nM Aβ. After 15 minutes of Aβ application to the patch membrane, on the other hand, extracellular applications of both neurotransmitters induced membrane currents, demonstrating the formation of pathways in the membrane capable of conducting ionic currents through the Aβ-containing pipette (Fig. 2A, lower traces). Additionally, the data show that Aβ induced perforated patches in a time-dependent manner making it possible to detect synaptic currents arising from synapses distant to the recording patch electrode (Fig. 2B). These results overwhelmingly show that Aβ is acting in a similar fashion to other pore forming peptides (i.e. gramicidin, amphotericin) well known to perforate neuronal membranes. Additionally, these novel results are appealing because they show that Aβ resembles other well known potent cytotoxic compounds, providing a novel molecular mechanism for neuronal toxicity of the Aβ peptide.

We next studied the current-voltage (I–V) relationships [21] to compare the ionic selectivity properties of perforates produced by Aβ (20 min of application) with those of gramicidin and amphotericin, known to form cationic and anionic selective pores, respectively. The application of GABA, the agonist for the GABA_A_ Cl^−^ current present in hippocampal neurons, showed that Aβ behaved like gramicidin, but not like amphotericin (Fig. 2C). For example, the Cl^−^ current recorded with Aβ in the pipette reversed direction near the expected equilibrium potential for Cl^−^ in the perforated mode [18]. Amphotericin, on the other hand, which dissipates the Cl^−^ gradient, reversed the GABA_A_ current at 0 mV. The data also shows that the AMPA current reversed close to 0 mV with the three perforating peptides (Fig. 2D), which is near the expected value for a non-selective cationic channel.

### Aβ action on conductance is not mediated by a classical channel-like behavior

**Figure 3:**
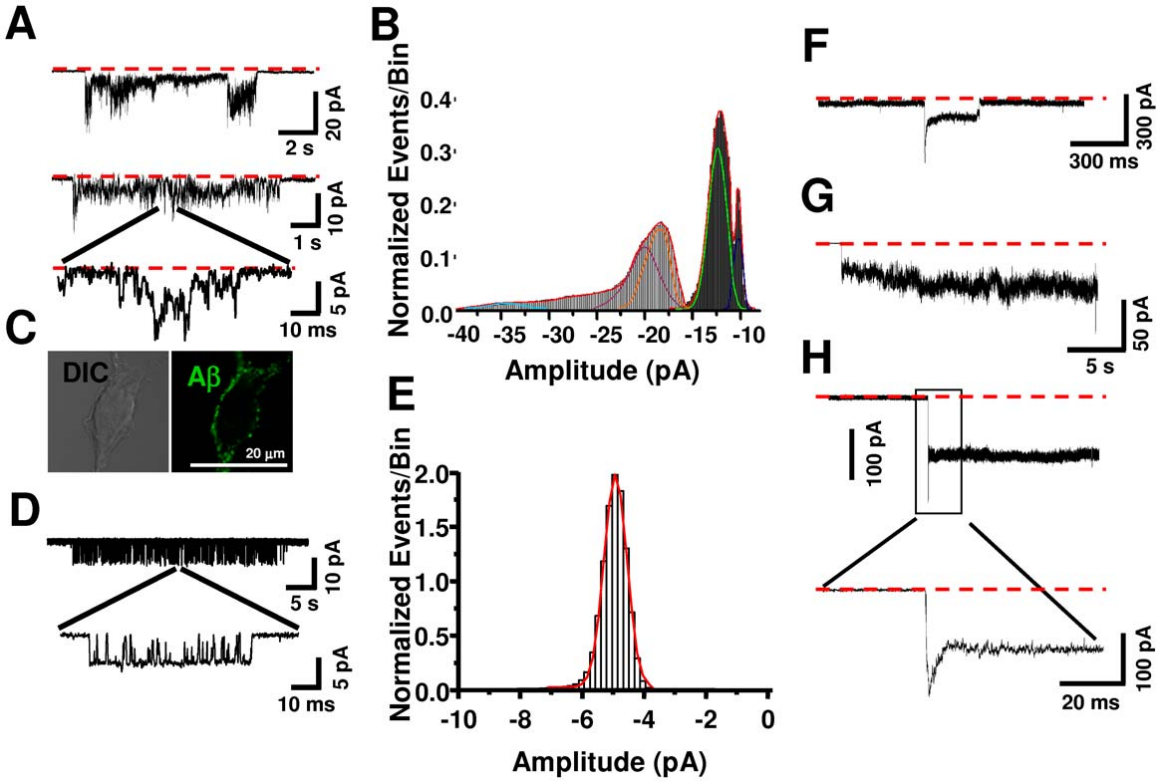
Aβ induced a non channel-like increase in microscopic membrane conductance. **A**, current traces show high sensitivity patch recordings obtained in the presence of 500 nM Aβ. The red line represents zero current. **B**, graph shows an all-point histogram obtained from the recordings in A. **C**, confocal micrograph shows the peripheral association of fluorescent Aβ to HEK cells. **D** current trace showing typical single channel behavior from a cell expressing alpha 1 human glycine receptors. Unlike Aβ the current expansion shows clear transitions between closed and open states. **E**, All point histogram fitted to a single conductance of 92 pS. **F**–**H**, traces show either partial or full membrane perforation in the presence of Aβ.

Some biophysical studies have indicated that Aβ can increase intracellular calcium and membrane conductance in artificial lipid bilayers and clonal cell lines [22]–[24], but demonstration of actual channel or pore formation in native brain membranes has been inadequately resolved. In our experiments, it was clear that Aβ was able to induce an increase in membrane noise before establishing the perforated configuration in hippocampal neurons. However, the noisy nature of the neuronal membrane related to activation of endogenous channels precluded us from studying the pore properties in more detail. To circumvent this, we recorded from HEK 293 cells before the formation of a perforated configuration. Recordings done in more than 40 cells did not show membrane events reminiscent of typical single channel behavior, in the sense of having well structured open and closing behavior, in the presence of Aβ. Therefore, the noisy nature of the microscopic current events produced by Aβ did not allow for a good discrimination between conformational states or to construct open and shut distributions (Fig. 3A). Nevertheless, plots of all-point current distributions from different patches showed multiple levels of peak conductance (200±2, 260±40, 360±60, 440±20 and 680±60 pS) supporting the occurrence of multiple membrane disruptions by Aβ (Fig. 3B) more than formation of a single unitary channel. In parallel experiments, we found that fluorescent Aβ was able to associate to cell membranes giving a morphological correlation to the membrane-perforating actions of the peptide (Fig. 3C). Furthermore, in some patches, Aβ produced a large transient increase in membrane current (1000–2000 pS) which we interpreted as spontaneous breakage-resealing of the membrane produced by Aβ that sometimes resulted in a whole cell configuration (Fig. 3F–H). Interestingly, Aβ was able to bind widely to HEK 293 cells and also caused the generation of a perforated configuration in these cells, as indicated by parallel monitoring of membrane capacitance (Fig. S3 and Fig. 3C). These data, therefore, indicate that Aβ affects the membrane inducing a range of current responses which are different from those of membrane channels, which have well defined conductance and time distributions due to their gating properties [25], [26]. For instance, the analysis of single channel currents associated with α1 glycine receptors provided a way of comparing a typical ion channel having a single channel conductance of 92±3 pS with Aβ activity (Fig. 3D,E). The previous experiments showed small and large microscopic membrane current events induced by Aβ. While the smaller Aβ perforations might exhibit a degree of ion selectivity (Fig. 2C), it is likely that the large ones might allow the entry of other molecules into the cell, which can be examined using fluorescent probes loaded in the pipette.

### The amyloid pore allows entry of a large molecule into neurons

**Figure 4:**
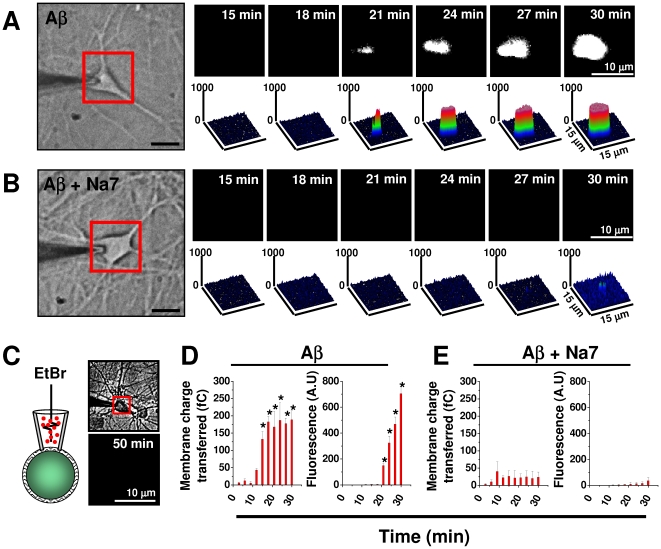
Aβ perforation causes entry of a small organic molecule in parallel with the increase in membrane conductance. **A**, the time dependent increase in cellular fluorescence associated with entry of ethidium bromide in presence of Aβ in the pipette. **B**, the effect of Aβ was blocked by the Na7 peptide. **C**, Ethidium bromide was unable to enter into the cell in the absence of Aβ. **D**–**E**, effect of Aβ on membrane current transferred and fluorescence in the absence and presence of Na7. * denotes a P<0.05 (n = 7–8 neurons).

The data in figure 4 show combined patch clamp-imaging recordings using patch pipettes filled with ethidium bromide in the presence and absence of Aβ (Fig. 4A,C). From this data, it is evident that a large (M.W. 394.3, ∼1.3 nm van der Waals diameter, PDB ID: 2ZOZ) organic molecule can enter the neuron in parallel with the process of electrical membrane perforation (Fig. 4A, D). In the absence of Aβ in the pipette (Fig. 4C), or with Na7, an Aβ-pore blocking peptide [27] (Fig. 4B,E), ethidium bromide was unable to enter into the cell. The size of this fluorescent molecule allows us to place a diameter of at least 1.5 nm for the large perforation induced by Aβ, which agrees with previous AFM data [10], [11]. Clearly, these large perforations might cause enormous homeostatic consequences for neuronal functions.

### The amyloid perforates can be inhibited by small peptides

**Figure 5:**
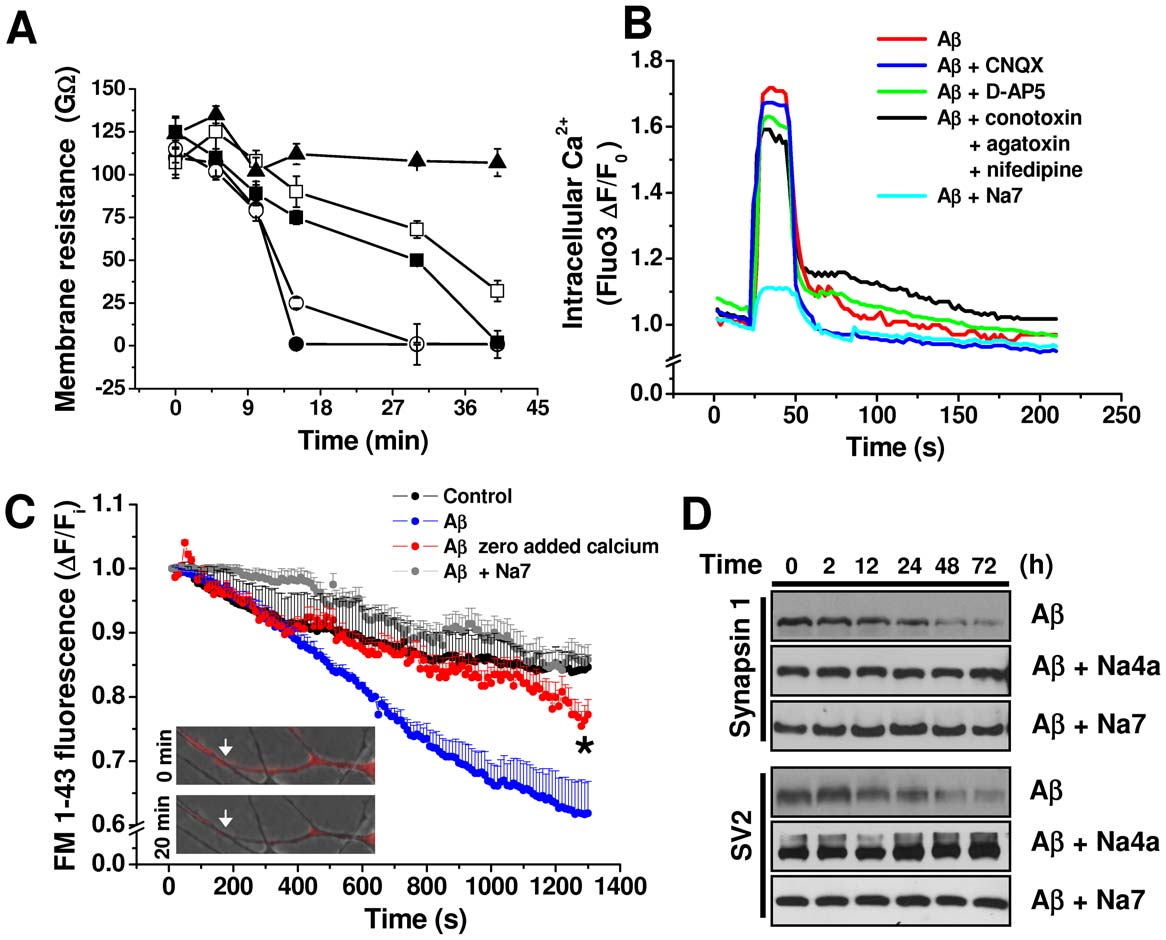
The effects of Aβ on membrane conductance and synaptotoxicity can be inhibited by small peptides. **A**, effect of Aβ(•) on membrane resistance in the absence and presence of Na7 (○ 1µM, ▪ 3 µM, □ 7 µM, ▴ 100 µM). **B**, effect of Aβ application on intracellular calcium increase and its inhibition by Na7, but not by other ion channel blockers. **C**, effects of Na7 and low calcium on Aβ-induced destaining of FM1–43. The insets show destaining of FM1–43 (arrows) at 20 min. **D**, time dependent reduction of synapsin and SV2 induced by Aβ and its inhibition by Na4a and Na7 (50 µM). Each point (mean ± SEM) was measured in a least 5 different hippocampal neurons.

One of the main issues related to the toxicity of Aβ in brain neurons is the identification of potential targets for the development of pharmaceutics capable of blocking its effects. In line with the idea that pore formation is relevant to Aβ toxic actions, it was reported that the increase in Ca^2+^ influx and lipid bilayer conductance was blocked by two small peptides [27]. Interestingly, these peptides also inhibited Aβ-induced cell death [28]. Furthermore, the increase in charge transfer and entry of ethidium bromide into the cell was well inhibited by this peptide (Fig. 4B,E). In addition, we found that the Na7 peptide produced a blockade of Aβ effects on membrane resistance (1/G) in hippocampal neurons in a concentration-dependent fashion (Fig. 5A). In experiments using hippocampal neurons loaded with fluo-4, Aβ produced a reversible increase in intracellular calcium, demonstrating the diffusible nature of Aβ. Additionally, this increase was antagonized by Na7 (Fig. 5B), but not by other blockers of ligand-gated or voltage-dependent calcium channels, suggesting that this effect was mainly mediated by Aβ-induced membrane perforation.

Consistent with the critical role of calcium in synaptic transmission and in agreement with recently published data [17], we found that 500 nM of Aβ enhanced the release of synaptic vesicles from hippocampal neurons. This synaptic facilitation was blocked by the presence of the Na7 peptide suggesting the participation of Aβ perforation in this phenomenon (Fig. 5C). Na7 and Na4a, another structurally related peptide (Fig. S1A), but not the inactive analogs Na13 and Na15 [29], also antagonized the delayed synaptotoxic effects of Aβ on synapsin I and SV2, two vesicular proteins (Fig. 5D), and in addition altered membrane charge and resistance (Fig. S1B,C). In conclusion, the data indicate that the perforating effects of Aβ are associated to microscopic structures resembling small fibrils (Fig. S2A), but not to unstructured forms of Aβ (Fig S2B). This data might be important for future pharmacological applications in terms of the neurotoxic activity of Aβ. Furthermore, these results strongly suggest that Aβ perforations are involved in synaptic dysfunction mediated by Aβ oligomers [17].

## Discussion

Only recent studies have dealt with the action of Aβ at concentrations without overt neurotoxicity on synaptic properties [3]. Although controversy exists on whether Aβ can up or down regulate specific components of synaptic transmission, several studies in rodent hippocampus showed that Aβ alters pre and postsynaptic components governing LTP, NMDA- and AMPA neurotransmissions and calcium homeostasis. No definitive mechanism is available to explain this variety of effects [30]–[32], hindering the development of anti Aβ therapies. On the other hand, due to the urgency of generating disease-modifying therapeutics to treat people suffering from AD, we believe that the present data provide novel insights into innovative strategies to interfere with the toxic processes likely initiated at the neuronal membrane level [29]. Future studies should decipher the characteristics of Aβ perforate formation in brain membranes. For example, although pore forming peptides have been in use for more than 40 years, most of their mechanisms for membrane insertion, pore formation and membrane conductance initiation have remained largely undetermined [13]. For example, from lipid bilayer studies, it was postulated that gramicidin required simultaneous insertion of two monomers on opposite faces of the lipid bilayer to perforate the membrane. However, this phenomenon might not occur in biological membranes. Our most recent experiments have shown that gramicidin forms oligomeric complex structures in aqueous solution and induces membrane perforations, similar to Aβ, rather than single channel currents in native cell membranes (unpublished results). Nevertheless, because Aβ can internalize rapidly [33], it might break the membrane inserting itself in both faces.

In agreement with the data in the present study, AFM and molecular dynamic studies of Aβ pores in bilayers support the presence of diverse, small and large molecular entities that possibly correspond to the functional perforation described in this study. The Aβ inner pore diameter appears to be much larger (at least 2.6 nm) than ion selective channels, which have an estimated diameter of 0.6 nm [21]. Overall, studies with AFM, molecular simulations and single channel conductances suggest a high range of pore sizes [34], and provide additional support to the idea that the phenomenon of insertion and conductance of Aβ are very complex. The proposal of a complex pore structure is consistent with a recent study that proposed a model for the Aβ pore [42]. Additionally, because these conducting Aβ entities appear to lack most regulatory mechanisms (i.e. post transductional modifications, inactivation, membrane anchoring, stable pore size) important for channel gating, we believe that they do not behave as classical ion channels to allow selective ion permeation. Since these membrane disruptions are important for neuronal toxicity, their blockade would be expected to inhibit synaptotoxicity, neurodegeneration and subsequently AD progression. Furthermore, this membrane permeabilization action of Aβ is in agreement with the vesicular depletion recently reported [17].

Interestingly, the actions of Aβ show strong similarities, although to a lesser extent, to the effect of α-latrotoxin (LTX) on neurotransmission [35]. For example, after a strong enhancement of synaptic transmission, LTX induced vesicle depletion and diminution in miniature potentials by a pore forming mechanism, having conductance and kinetic properties very similar to those of pores formed by Aβ in lipid bilayers [14].

**Figure 6:**
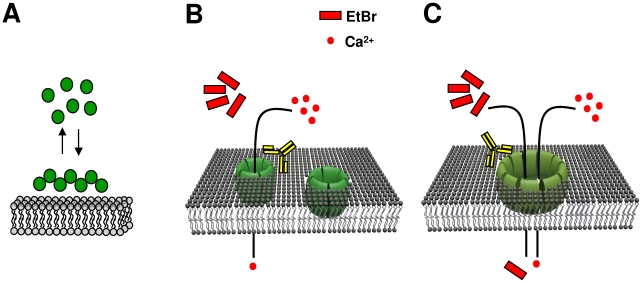
The scheme is a simplified model for association, micro and macro perforation induced by Aβ in cellular membranes. **A**, aggregation and binding (association) of Aβ to the neuronal membrane **B**, smaller perforations are associated to a selective ion influx (gramicin-like ion influx). **C**, larger perforations allow the entry of large molecules, which include EtBr (∼1.3 nm). All these Aβ effects are blocked by application of anti- Aβ antibody.

In summary, our working model to explain the toxicity of Aβ in Alzheimer's disease proposes the existence of diverse membrane structures that can progress from a small, ion selective pore, to a large membrane perforation (Fig. 6). All these Aβ perforations are capable of producing a wide range of toxic effects ranging from synaptotoxicity to cell death.

## Materials and Methods

### Ethics Statement

All animals were handled in strict accordance with the Animal Welfare Assurance (permit number 2008100A) and all animal work was approved by the appropriate Ethics and Animal Care and Use Committee of the University of Concepcion.

### Cultures

Hippocampal neurons were obtained from 18 day pregnant mouse embryos (C57BL/J6) or Sprague-Dawley rat embryos as previously described [36] in accordance with NIH recommendations. Human Embryonic Kidney 293 cells (HEK) were cultivated in D-MEM (Dulbecco's Modified Eagle Medium, Life Technologies, Inc. USA) supplemented with 10% fetal bovine serum (Life Technologies Inc. USA.) and streptomycin-penicillin (200 units each, Life Technologies Inc. USA). Cells were maintained with 5% CO_2_ at 37°C. HEK 293 cells were kindly provided by Dr. Olate (University of Concepcion) and have been previously described in the lab [41].

### Amyloid Aggregation

Human Aβ_1–40_ labeled with Rhodamine Green at its N-terminus and unlabeled were purchased from Anaspec (CA, USA) and Tocris (MO, USA), respectively. Aβ_1–40_ was dissolved in DMSO (10 mg/ml) and stored in aliquots at −20°C. For the preparation of Aβ aggregates (80 µM), aliquots of peptide stock (250 µg in 25 µl of DMSO) were added to 700 µl of PBS (Gibco, USA) and continuously agitated (200 RPM at 37°C) for 90 minutes and stored at 4°C. Aβ_1–40_ Rhodamine Green (Abs/Em = 502/527 nm) was dissolved in DMSO (4 mg/ml) and immediately stored in aliquots at −20°C.

### Recordings

Patch pipettes having a resistance between 1 and 3 MΩ were prepared from filament-containing borosilicate micropipettes. Currents were measured with the whole-cell patch-clamp technique at a holding potential of −60 mV using an Axopatch 200B (molecular devices, USA) amplifier as previously described [37], [38]. Perforated recordings were obtained as follows: the perforating agent was added into the pipette solution and a 5 mV pulse was used to monitor the formation of the perforation. Gramicidin and amphotericin were used at 100 µg/ml. Short applications of Aβ, GABA (100 µM) and AMPA (100 µM) were done via lateral motion of a multi-pipette array (approx. 200 µm in diameter). Some experiments involved an external solution without added calcium, Na7 (20–100 µM), Na4a (20 µM) or the inactive peptides Na13 and Na15 (20 µM).

### Western Blots

Standard Western blotting procedures were followed. Equal amounts of protein were separated on 10% SDS-PAGE gels. Protein bands were transferred onto nitrocellulose membranes, blocked with 5% milk and incubated with a primary antibody using the following concentrations: anti-Aβ (NAB228, Santa Cruz Biotechnology, CA, USA) 1∶500, anti-Synapsin I (AB1543, Chemicon, MA, USA) 1∶1000, anti-SV2 (Developmental Studies Hybridoma Bank, IA, USA) 1∶200. Immunoreactive bands were visualized with ECL plus Western Blotting Detection System (PerkinElmer, MA, USA).

### Intracellular Calcium Imaging

Neurons were loaded with Fluo-4 AM (1 µM in pluronic acid/DMSO, Molecular Probes, Eugene, OR, USA) for 30 min at 37°C. The neurons were then washed twice with external solution and incubated for 30 min at 37°C. The cells were mounted in a perfusion chamber that was placed on the stage of an inverted fluorescent microscope (Eclipse TE, Nikon, USA). The cells were briefly (200 ms) illuminated using a computer-controlled Lambda 10-2 filter wheel (Sutter Instruments, USA). Regions of interest (ROI) were marked in a field having usually more than 10 cells. Images were collected at 2–5 s intervals during a continuous 5-min period. The imaging was carried out with a SensiCam camera (PCO, Germany) using Axon Instruments Workbench 2.2 software. The calcium channel inhibitors used were conotoxin (1 µM), agatoxin (1 µM), nifedipine (3 µM), CNQX (4 µM) and D-AP5 (50 µM).

### FM1-43 Loading and Unloading

Presynaptic vesicles were labeled by exposure to FM1-43 (15 µM, Molecular Probes, USA) during a high-K^+^ depolarization for 5 min and immediately washed, as previously described [39], [40]. Coverslips were mounted on a rapid switching flow perfusion chamber with an inverted fluorescent microscope (Eclipse TE, Nikon, USA) equipped with a 100× objective (oil immersion, NA 1.4). Depolarization-dependent destaining was induced by bath perfusion with 30 mM K^+^ (equiosmolar replacement of Na^+^).

### Immunocytochemistry

Hippocampal neurons treated during 15 minutes with 500 nM fluorescent Aβ were fixed for 15 min with 4% paraformaldehyde and permeabilized with 0.1% triton X-100 in PBS and incubated with anti-MAP2 1∶300 (Santa Cruz Biotechnology, CA, USA). Secondary anti-rabbit IgG (Jackson ImmunoResearch Laboratories, PA) conjugated with Cy3 was used at 1∶500 for 2 hours.

### Calculation of Ethidium Bromide Size

The van der Waals diameter of EtBr was measured with Swiss PDBviewer using atomic coordinates for the crystal structure of the ethidium-bound form of the multi-drug binding transcriptional repressor CgmR (PDB ID: 2ZOZ).

### Data Analysis

Non-lineal analysis was performed using Origin (Microcal). Membrane charge was analyzed by integrating the transient capacitative current after subtracting the pipette capacitance. The values are expressed as mean ± SEM (standard error mean). Statistical differences were determined using Student's t test or ANOVA. The experiments were performed in triplicate.

## Supporting Information

Figure S1 Blockade of Aβ induced membrane disruption by small peptides. A, sequence of Aβ and mini peptides used in this study (NA7, NA4a, NA13 and NA15). B–C, shows the effect of Aβ (500 nM) and Aβ plus mini peptides (20 µM) on the transferred membrane charge and resistance, respectively. The bars are means ±SEM. * denotes a P<0.05.
(0.80 MB TIF)

Figure S2 Perforating actions of Aβ were associated to the presence of fibril-like structures. A, the upper electron micrograph shows active structures labeled with 5 nm gold-particles. B, the current trace show that these structures caused membrane perforations in rat hippocampal neurons. Lower panels show a more globular Aβ structure that was found to be inactive. Data is typical from 6 experiments.
(2.18 MB TIF)

Figure S3 Aβ induce membrane perforations in HEK293 cell. A, The confocal micrograph shows the peripherical association of fluorescent Aβ to HEK cells (30 min). B, capacitative membrane currents were recorded using a cell-attached configuration at the beginning (0 min) and after 30 min of Aβ application via the patch pipette. C, effects of Aβ on the transferred membrane charge induced by 5 mV depolarization pulse in HEK cells, hippocampal and cortical neurons. Each point (mean ± SEM) was measured in a least 6 different cells.
(1.58 MB TIF)

## References

[1] Selkoe DJ (2002). Alzheimer's disease is a synaptic failure.. Science.

[2] Matsuoka Y, Gray AJ, Hirata-Fukae C, Minami SS, Waterhouse EG (2007). Intranasal NAP administration reduces accumulation of amyloid peptide and tau hyperphosphorylation in a transgenic mouse model of Alzheimer's disease at early pathological stage. (Translated from eng). J Mol Neurosci.

[3] Haass C, Selkoe DJ (2007). Soluble protein oligomers in neurodegeneration: lessons from the Alzheimer's amyloid beta-peptide.. Nat Rev Mol Cell Biol.

[4] Soto C, Estrada L (2005). Amyloid inhibitors and beta-sheet breakers.. Subcell Biochem.

[5] Wang Q, Walsh DM, Rowan MJ, Selkoe DJ, Anwyl R (2004). Block of long-term potentiation by naturally secreted and synthetic amyloid beta-peptide in hippocampal slices is mediated via activation of the kinases c-Jun N-terminal kinase, cyclin-dependent kinase 5, and p38 mitogen-activated protein kinase as well as metabotropic glutamate receptor type 5.. J Neurosci.

[6] Garrido JL, Godoy JA, Alvarez A, Bronfman M, Inestrosa NC (2002). Protein kinase C inhibits amyloid beta peptide neurotoxicity by acting on members of the Wnt pathway.. Faseb J.

[7] Maccioni RB, Otth C, Concha II, Munoz JP (2001). The protein kinase Cdk5. Structural aspects, roles in neurogenesis and involvement in Alzheimer's pathology.. Eur J Biochem.

[8] Daniels WM, Hendricks J, Salie R, Taljaard JJ (2001). The role of the MAP-kinase superfamily in beta-amyloid toxicity.. Metab Brain Dis.

[9] Arispe N, Pollard HB, Rojas E (1994). The ability of amyloid beta-protein [A beta P (1–40)] to form Ca2+ channels provides a mechanism for neuronal death in Alzheimer's disease.. Ann N Y Acad Sci.

[10] Lin H, Bhatia R, Lal R (2001). Amyloid beta protein forms ion channels: implications for Alzheimer's disease pathophysiology.. Faseb J.

[11] Jang H, Zheng J, Nussinov R (2007). Models of beta-amyloid ion channels in the membrane suggest that channel formation in the bilayer is a dynamic process.. Biophys J.

[12] Quist A, Doudevski I, Lin H, Azimova R, Ng D (2005). Amyloid ion channels: a common structural link for protein-misfolding disease.. Proc Natl Acad Sci U S A.

[13] Andersen OS, Koeppe RE, Roux B (2005). Gramicidin channels.. IEEE Trans Nanobioscience.

[14] Orlova EV, Rahman MA, Gowen B, Volynski KE, Ashton AC (2000). Structure of alpha-latrotoxin oligomers reveals that divalent cation-dependent tetramers form membrane pores.. Nat Struct Biol.

[15] Ebihara S, Shirato K, Harata N, Akaike N (1995). Gramicidin-perforated patch recording: GABA response in mammalian neurones with intact intracellular chloride.. J Physiol.

[16] Tajima Y, Ono K, Akaike N (1996). Perforated patch-clamp recording in cardiac myocytes using cation-selective ionophore gramicidin.. Am J Physiol.

[17] Parodi J, Sepulveda FJ, Roa J, Opazo C, Inestrosa NC (2010). β-amyloid causes depletion of synaptic vesicles leading to neurotransmission failure.. J Biol Chemn.

[18] Hamill OP, Marty A, Neher E, Sakmann B, Sigworth FJ (1981). Improved patch-clamp techniques for high-resolution current recording from cells and cell-free membrane patches.. Pflugers Arch.

[19] Le Foll F, Castel H, Soriani O, Vaudry H, Cazin L (1998). Gramicidin-perforated patch revealed depolarizing effect of GABA in cultured frog melanotrophs.. J Physiol.

[20] Rhee JS, Ebihara S, Akaike N (1994). Gramicidin perforated patch-clamp technique reveals glycine-gated outward chloride current in dissociated nucleus solitarii neurons of the rat.. J Neurophysiol.

[21] Hille B (2001). Ion channels of excitable membranes,.

[22] Demuro A, Mina E, Kayed R, Milton SC, Parker I (2005). Calcium dysregulation and membrane disruption as a ubiquitous neurotoxic mechanism of soluble amyloid oligomers.. J Biol Chem.

[23] Alarcon JM, Brito JA, Hermosilla T, Atwater I, Mears D (2006). Ion channel formation by Alzheimer's disease amyloid beta-peptide (Abeta40) in unilamellar liposomes is determined by anionic phospholipids.. Peptides.

[24] Kawahara M, Kuroda Y, Arispe N, Rojas E (2000). Alzheimer's beta-amyloid, human islet amylin, and prion protein fragment evoke intracellular free calcium elevations by a common mechanism in a hypothalamic GnRH neuronal cell line.. J Biol Chem.

[25] Beato M, Groot-Kormelink PJ, Colquhoun D, Sivilotti LG (2004). The activation mechanism of alpha1 homomeric glycine receptors.. J Neurosci.

[26] Morales A, Nguyen QT, Miledi R (1994). Electrophysiological properties of newborn and adult rat spinal cord glycine receptors expressed in Xenopus oocytes.. Proc Natl Acad Sci U S A.

[27] Arispe N (2004). Architecture of the Alzheimer's A beta P ion channel pore.. J Membr Biol.

[28] Arispe N, Diaz JC, Simakova O (2007). Abeta ion channels. Prospects for treating Alzheimer's disease with Abeta channel blockers.. Biochim Biophys Acta.

[29] Diaz JC, Linnehan J, Pollard H, Arispe N (2006). Histidines 13 and 14 in the Abeta sequence are targets for inhibition of Alzheimer's disease Abeta ion channel and cytotoxicity.. Biol Res.

[30] Glabe CG, Kayed R (2006). Common structure and toxic function of amyloid oligomers implies a common mechanism of pathogenesis.. Neurology.

[31] Snyder EM, Nong Y, Almeida CG, Paul S, Moran T (2005). Regulation of NMDA receptor trafficking by amyloid-beta.. Nat Neurosci.

[32] Stephan A, Laroche S, Davis S (2001). Generation of aggregated beta-amyloid in the rat hippocampus impairs synaptic transmission and plasticity and causes memory deficits.. J Neurosci.

[33] Kandimalla KK, Scott OG, Fulzele S, Davidson MW, Poduslo JF (2009). Mechanism of neuronal versus endothelial cell uptake of Alzheimer's disease amyloid beta protein.. PLoS One.

[34] Kourie JI, Henry CL, Farrelly P (2001). Diversity of amyloid beta protein fragment [1–40]-formed channels.. Cell Mol Neurobiol.

[35] Ashton AC, Volynski KE, Lelianova VG, Orlova EV, Van Renterghem C (2001). alpha-Latrotoxin, acting via two Ca2+-dependent pathways, triggers exocytosis of two pools of synaptic vesicles.. J Biol Chem.

[36] Tapia JC, Mentis G, Navarrete R, Nualart F, Figueroa E (2001). Early expression of glycine and GABAA receptors in developing spinal cord neurons: effects on neurite outgrowth.. Neuroscience.

[37] Sepúlveda FJ, Opazo C, Aguayo LG (2009). Alzheimer beta-amyloid blocks epileptiform activity in hippocampal neurons.. Mol Cell Neurosci.

[38] Pancetti F, Oyarce M, Aranda M, Parodi J, Aguayo LG (2004). S-methylcysteine may be a causal factor in monohalomethane neurotoxicity.. Neurotoxicology.

[39] Ryan TA, Smith SJ, Reuter H (1996). The timing of synaptic vesicle endocytosis.. Proc Natl Acad Sci U S A.

[40] Ryan TA, Reuter H, Wendland B, Schweizer FE, Tsien RW (1993). The kinetics of synaptic vesicle recycling measured at single presynaptic boutons.. Neuron.

[41] Yevenes GE, Peoples RW, Tapia JC, Parodi J, Soto X (2003). Modulation of glycine-activated ion channel function by G protein subunits.. Nat Neurosci.

[42] Jang H, Arce FT, Capone R, Ramachandran S, Lal R (2010). Misfolded amyloid ion channels present mobile beta-sheet subunits in contrast to conventional ion channels.. Biophys J.

