# Supplementary figures and images for: Synaptotoxicity of Alzheimer Beta Amyloid Can Be Explained by Its Membrane Perforating Property
Source: PLoS One. 2010 Jul 27;5(7):e11820. doi: 10.1371/journal.pone.0011820 (PMC2910737; doi:10.1371/journal.pone.0011820)

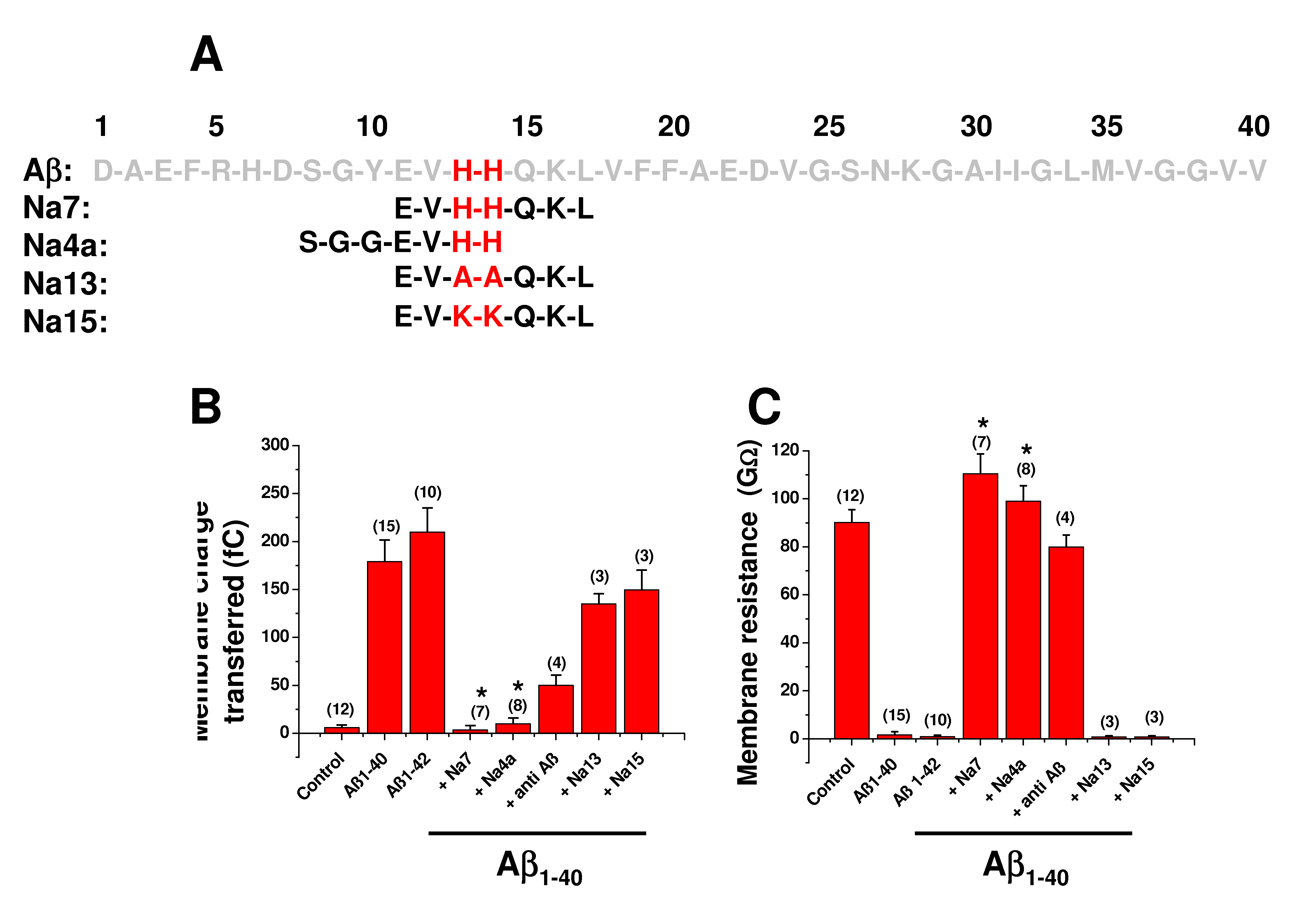

Supplement: Figure S1 — Blockade of Aβ induced membrane disruption by small peptides. A, sequence of Aβ and mini peptides used in this study (NA7, NA4a, NA13 and NA15). B–C, shows the effect of Aβ (500 nM) and Aβ plus mini peptides (20 µM) on the transferred membrane charge and resistance, respectively. The bars are means ±SEM. * denotes a P<0.05. (0.80 MB TIF) [file pone.0011820.s001.tif]

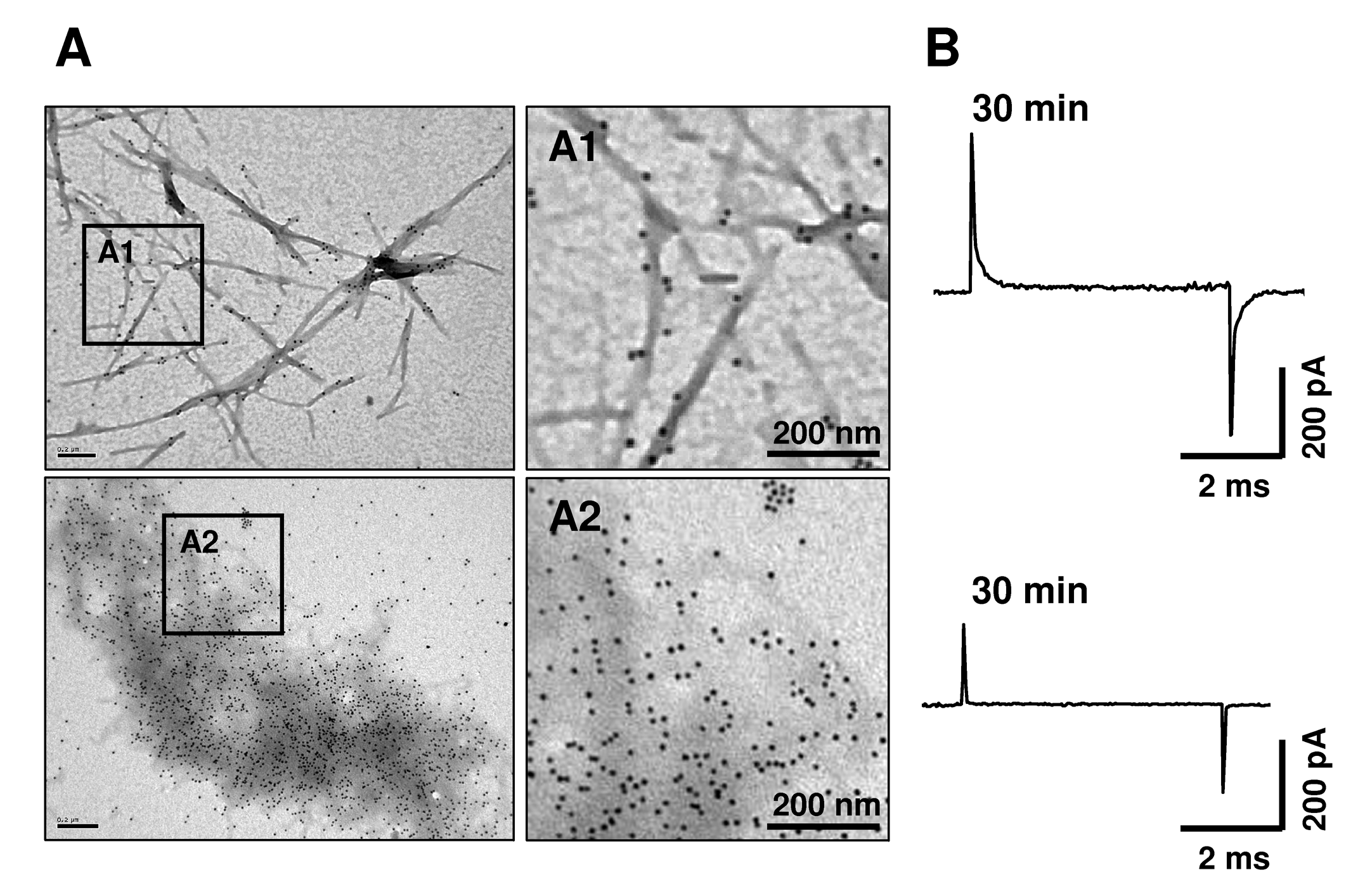

Supplement: Figure S2 — Perforating actions of Aβ were associated to the presence of fibril-like structures. A, the upper electron micrograph shows active structures labeled with 5 nm gold-particles. B, the current trace show that these structures caused membrane perforations in rat hippocampal neurons. Lower panels show a more globular Aβ structure that was found to be inactive. Data is typical from 6 experiments. (2.18 MB TIF) [file pone.0011820.s002.tif]

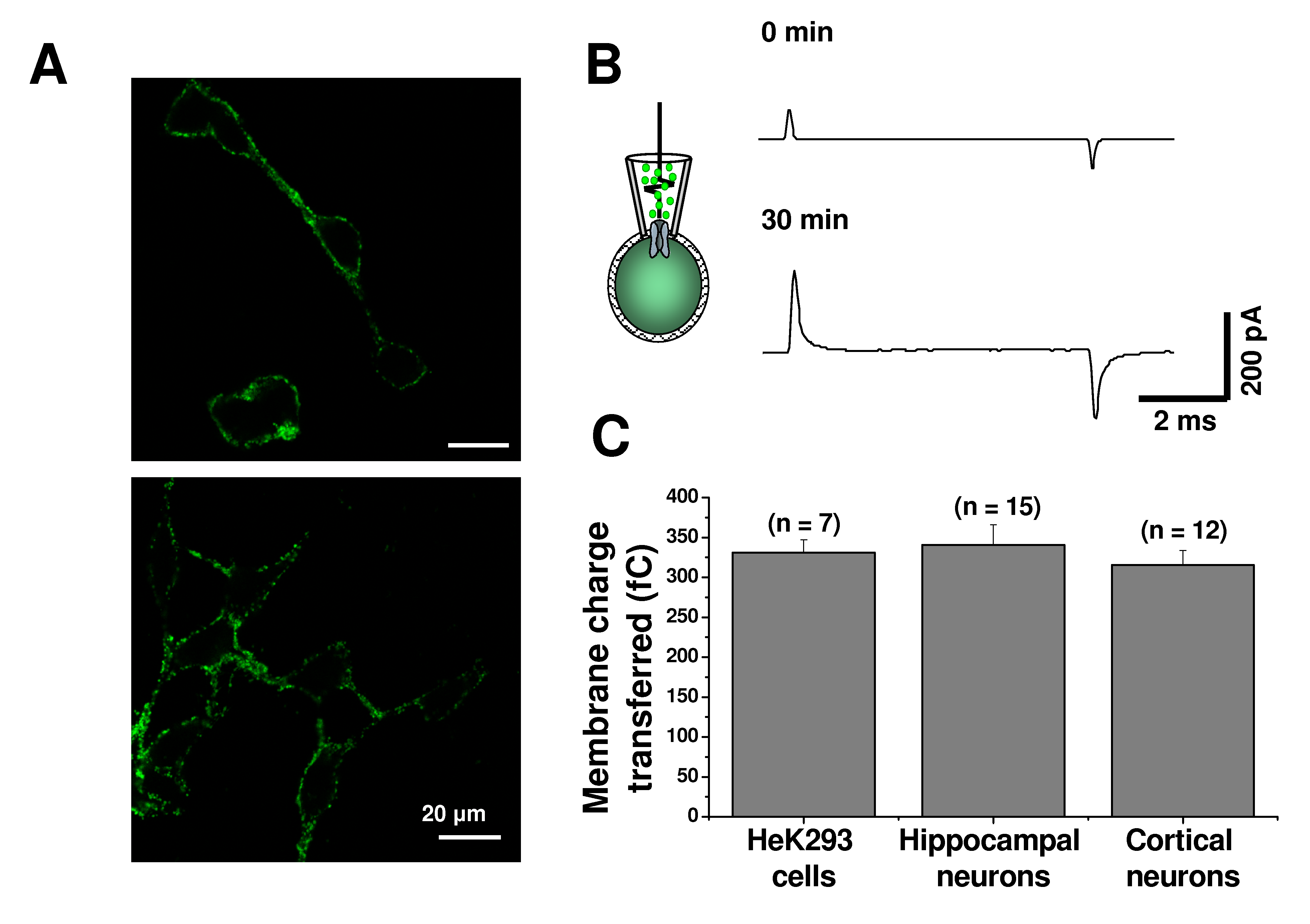

Supplement: Figure S3 — Aβ induce membrane perforations in HEK293 cell. A, The confocal micrograph shows the peripherical association of fluorescent Aβ to HEK cells (30 min). B, capacitative membrane currents were recorded using a cell-attached configuration at the beginning (0 min) and after 30 min of Aβ application via the patch pipette. C, effects of Aβ on the transferred membrane charge induced by 5 mV depolarization pulse in HEK cells, hippocampal and cortical neurons. Each point (mean ± SEM) was measured in a least 6 different cells. (1.58 MB TIF) [file pone.0011820.s003.tif]
